# Retention of fatty acyl desaturase 1 (*fads1*) in Elopomorpha and Cyclostomata provides novel insights into the evolution of long-chain polyunsaturated fatty acid biosynthesis in vertebrates

**DOI:** 10.1186/s12862-018-1271-5

**Published:** 2018-10-19

**Authors:** Mónica Lopes-Marques, Naoki Kabeya, Yu Qian, Raquel Ruivo, Miguel M. Santos, Byrappa Venkatesh, Douglas R. Tocher, L. Filipe C. Castro, Óscar Monroig

**Affiliations:** 1 Interdisciplinary Centre of Marine and Environmental Research (CIIMAR/CIMAR), U. Porto – University of Porto, Terminal de Cruzeiros do Porto de Leixões, Av. General Norton de Matos s/n, 4450-208 Matosinhos, Portugal; 2 Laboratory of Histology and Embryology, Department of Microscopy, Institute of Biomedical Sciences Abel Salazar (ICBAS), U.Porto – University of Porto, Rua Jorge Viterbo Ferreira 228, P 4050-313 Porto, Portugal; 3 Department of Aquatic Bioscience, Graduate School of Agricultural and Life Sciences, The University of Tokyo, 1-1-1, Yayoi, Bunkyo-ku, Tokyo, 113-8657 Japan; 4 Institute of Aquaculture, Faculty of Natural Sciences, University of Stirling, Stirling, FK9 4LA Scotland, UK; 5 Faculty of Sciences (FCUP), Department of Biology, U.Porto – University of Porto, Rua do Campo Alegre, P 4169-007 Porto, Portugal; 6 Comparative Genomics Laboratory, Institute of Molecular and Cell Biology, A*STAR (Agency for Science, Technology and Research), Biopolis, Singapore, 138673 Singapore; 7 Instituto de Acuicultura Torre de la Sal (IATS-CSIC), Ribera de Cabanes, 12595 Castellón, Spain

**Keywords:** Biosynthesis, Fatty acyl desaturase, Gene duplication, Gene loss, Long-chain polyunsaturated fatty acids

## Abstract

**Background:**

Provision of long-chain polyunsaturated fatty acids (LC-PUFA) in vertebrates occurs through the diet or via endogenous production from C_18_ precursors through consecutive elongations and desaturations. It has been postulated that the abundance of LC-PUFA in the marine environment has remarkably modulated the gene complement and function of Fads in marine teleosts. In vertebrates two fatty acyl desaturases, namely *Fads1* and *Fads2*, encode ∆5 and ∆6 desaturases, respectively. To fully clarify the evolutionary history of LC-PUFA biosynthesis in vertebrates, we investigated the gene repertoire and function of *Fads* from species placed at key evolutionary nodes.

**Results:**

We demonstrate that functional *Fads1*Δ5 and *Fads2*∆6 arose from a tandem gene duplication in the ancestor of vertebrates, since they are present in the Arctic lamprey*.* Additionally, we show that a similar condition was retained in ray-finned fish such as the Senegal bichir and spotted gar, with the identification of *fads1* genes in these lineages. Functional characterisation of the isolated desaturases reveals the first case of a Fads1 enzyme with *∆5* desaturase activity in the Teleostei lineage, the Elopomorpha. In contrast, in Osteoglossomorpha genomes, while no *fads1* was identified, two separate *fads2* duplicates with ∆6 and ∆5 desaturase activities respectively were uncovered.

**Conclusions:**

We conclude that, while the essential genetic components involved LC-PUFA biosynthesis evolved in the vertebrate ancestor, the full completion of the LC-PUFA biosynthesis pathway arose uniquely in gnathostomes.

**Electronic supplementary material:**

The online version of this article (10.1186/s12862-018-1271-5) contains supplementary material, which is available to authorized users.

## Background

Long-chain (≥C_20_) polyunsaturated fatty acids (LC-PUFA) and their derivatives are biologically active molecules that are involved in neural function, signalling and regulation of lipid metabolism, inflammation and cell division [1]. Among LC-PUFA, arachidonic acid (ARA, 20:4n-6), eicosapentaenoic acid (EPA, 20:5n-3) and docosahexaenoic acid (DHA, 22:6n-3) play particularly important roles in the abovementioned physiological processes [2, 3]. In vertebrates, biosynthesis of LC-PUFA such as ARA, EPA and DHA is achieved by sequential reactions towards the dietary essential fatty acids (EFA) linoleic acid (LA, 18:2n-6) and α-linolenic acid (ALA, 18:3n-3), which are catalysed by fatty acyl desaturase (Fads) and elongation of very long-chain fatty acid (Elovl) enzymes [3]. Briefly, ARA and EPA are synthesised from LA and ALA, respectively, by two distinct pathways called the “Δ6 pathway” (Δ6 desaturation – elongation – Δ5 desaturation) or the “Δ8 pathway” (elongation – Δ8 desaturation – Δ5 desaturation). Moreover, DHA biosynthesis generally proceeds through the so-called “Sprecher pathway”, comprising two consecutive elongation steps from EPA to produce 24:5n-3, which is then Δ6 desaturated to 24:6n-3 prior to being converted to DHA by partial β-oxidation in peroxisomes [4, 5]. Interestingly, an alternative pathway involving direct Δ4 desaturation of 22:5n-3 to DHA has been described in teleosts [6, 7] and, more recently, found to operate in mammalian cells [8].

In mammals, Δ5 and Δ6 desaturation reactions are specifically catalysed by FADS1 and FADS2 enzymes, respectively [2]. Orthologues of both *fads1*Δ5 and *fads2*Δ6 were previously identified in the cartilaginous fish *Scyliorhinus canicula*, an indication that they emerged before gnathostome origin [9]. Furthermore, while mammals, birds, reptiles and amphibians also possess *fads1* and *fads2* genes, an orthologue of *fads1* has not be identified to date in Teleostei [3, 9]. In addition, the repertoire of Teleostei *fads2* varies significantly among lineages, with some species possessing one (e.g. *Danio rerio*), two (e.g. *Monopterus albus*), three (e.g. *Oreochromis niloticus*) or four (e.g. *Salmo salar*) *fads2* paralogues, whereas others lack completely *fads* genes in their genomes (e.g. *Tetraodon nigroviridis*) [3]. With regards to function, many Teleostei Fads2 retain the Δ6 desaturase phenotype but, interestingly, they also exhibit a more varied spectrum of activities including bifunctional Δ6Δ5 desaturase [10–13] and Δ4 desaturase [7] as a result of a functionalisation process hypothesised to have occurred in response to dietary availability in natural prey [9].

Although the gene complement and functions of *fads* are well understood in Chondrichthyes, numerous Teleostei species and Tetrapoda [2, 3], the lack of information in lineages such as Cyclostomata, Polypteriformes, Holostei and post 3R lineages (e.g. Elopomorpha and Osteoglossomorpha) hampers the full comprehension of FADS function in vertebrates. Hence, to fully clarify the history of LC-PUFA biosynthesis in vertebrates, we isolated and functionally characterised the *fads* complement from species placed at key phylogenetic nodes, i.e. the cyclostome *Lethenteron camtschaticum* (Arctic lamprey), representative of the most ancient lineage of extant vertebrates, and four species of ray-finned fish. Among the latter, we investigated two species that diverged before the teleost specific whole genome duplication (3R WGD), namely the Polypteriforme, *Polypterus senegalus* (Senegal bichir) and the Lepisosteiforme, *Lepisosteus oculatus* (spotted gar) [14], and two others that diverged after the teleost specific 3R whole genome duplication (WGD), namely the Elopomorpha, *Anguilla japonica* (Japanese eel) and the Osteoglossomorpha, *Pantodon buchholzi* (African butterfly fish) [15, 16]. Our findings provide a definitive understanding of the evolutionary history of key components of essential LC-PUFA biosynthesis and demonstrate that functional *fads1*Δ5 and *fads2*Δ6 emerged in the vertebrate ancestor.

## Methods

### Sequence collection and phylogenetic analysis

**Fig. 1:**
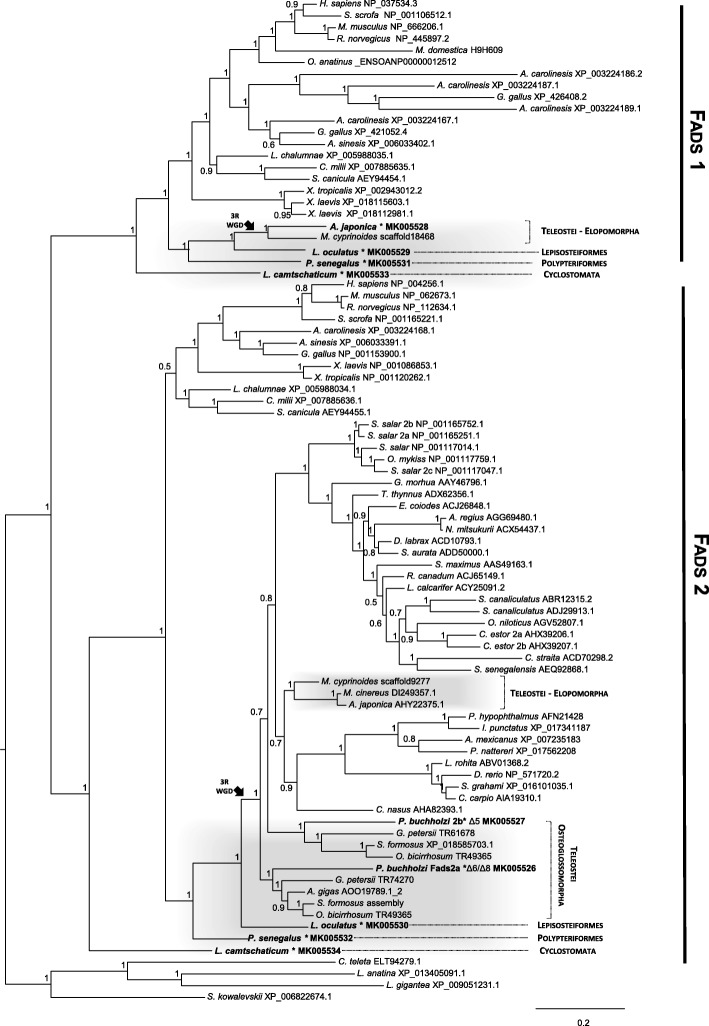
Maximum likelihood phylogenetic analysis of FADS1 and FADS2 amino acid sequences. Values at nodes indicate posterior probabilities, * indicates FADS isolated and functionally analysed in this work. Black arrow (3R WGD) approximates the timing of the teleost duplication. Accession numbers are indicated

The initial sequence collection for phylogenetic analysis was performed through blastp and blastn searches in NCBI, using as query *Homo sapiens* FADS1 (NP_037534.3) and FADS2 (NP_004256.1) sequences. Additionally, to ensure a full collection of *fads* sequences in Teleostei a second search was performed in NCBI targeting specifically the nucleotide collection and non-redundant protein database. From these results a set of Fads amino acid sequences representative of the major vertebrate clades was collected for phylogenetic analysis (Accession numbers provided in Fig. 1). Searches revealed that the *Scleropages formosus* presented a 3’partial Fads-like sequence (XP_018598908.1); this sequence was completed by performing blastn searches in *S. formosus* transcriptome SRA reads (NCBI accession: SRX1668426 to 32). Additionally, *Gnathonemus petersii* and *Osteoglossum bicirrhosum fads*-like genes were assembled from their genomic SRA (SRX2235994 and 95) with Geneious V 7.1.9 using as reference the previously curated *S. formosus fads.* The 86 sequences from the databases and 9 sequences functionally characterised in the present study were aligned with MAFFT v7.306 [17]. The best alignment method was determined automatically resulting in L-INS-i method [18]. Columns containing 90% gaps were stripped from sequence alignment leaving a total of 451 sites for phylogenetic analysis. Sequence alignment was then submitted to PhyML v3.0 server [19] for Maximum Likelihood (ML) phylogenetic evolutionary model and was automatically selected by smart model selection SMS resulting in LG + G + I, and branch support was calculated using Abayes [20]. The resulting tree was visualised using FigTree v1.3.1 (http://tree.bio.ed.ac.uk/software/figtree/) and rooted with invertebrate Fads sequences.

### Isolation of *fads* for functional characterisation in yeast

Selected Fads from species within major pre and post 3R WGD lineages were functionally characterised by expressing their ORF in yeast *S. cerevisiae*. In order to isolate the corresponding *fads* ORF sequences, initial tblastn searches using *S. canicula fads1* (AEY94454) *and fads2* (AEY94455) as queries were carried out in the genome assembly of *L. camtschaticum* (https://www.ncbi.nlm.nih.gov/assembly/GCA_000466285.1) and *A. japonica* (GCA_000470695), and transcriptome SRA from *P. senegalus* (SRX796491, SRX732875) and *P. buchholzi* (SRX666400). The resulting hits were assembled into predicted full-length ORF using the corresponding bait sequences as references. With *L. oculatus*, although annotations of *fads1-* and *fads2-*like genes were available at GenBank (XM_015338726 for *fads1*) and Ensembl (ENSLOCG00000007048 and ENSLOCG00000007031 for *fads1* and *fads2*, respectively), these gene predictions were poor due to the genome coverage. Therefore, we performed manual mapping of the genomic sequence (NC_023205) using transcript sequences of *fads1-* and *fads2-*like genes retrieved from their transcriptome SRA (SRX661022). The obtained transcripts were then assembled into full-length ORF sequences referring to the genomic sequence. All final assembled/predicted *fads* ORF sequences were then used as reference to design primers for further cloning into the yeast expression vector pYES2 (Thermo Fisher Scientific, Waltham, MA, USA). Briefly, cDNA prepared from total RNA extracted from *L. camtschaticum*, *P. senegalus*, *L. oculatus*, *P. buchholzi* and *A. japonica* was used to amplify the corresponding *fads* ORF by PCR using primers containing restriction sites at both the start and stop codon (see Additional file 1 for primer details and PCR conditions). Each PCR product was digested with the corresponding restriction enzymes and ligated into similarly restricted pYES2 [6, 10]. All pYES2 clones were confirmed by sequencing (GATC Biotech Constance, Germany) prior to being used in functional assays in yeast.

### Yeast expression assays

Yeast transformation with pYES2 and the culture of yeast *S. cerevisiae* were carried out as described previously [10, 21]. The resulting transgenic yeast expressing each *fads* were grown in the presence of PUFA including Δ6 (18:3n-3 and 18:2n-6), Δ8 (20:2n-6 and 20:3n-3), Δ5 (20:4n-3 and 20:3n-6) and Δ4 (22:5n-3 and 22:4n-6) desaturase substrates. The PUFA substrates were exogenously supplemented at final concentrations of 0.5 mM (C_18_), 0.75 mM (C_20_) and 1.0 mM (C_22_) to compensate for decreased efficiency of uptake with increased chain length [21]. All FA substrates (98–99% pure), except for 18:4n-3 and 20:4n-3, were purchased from Nu-Chek Prep, Inc. (Elysian, MN, USA). Moreover, 18:4n-3 and 20:4n-3 were obtained from Sigma-Aldrich (St Louis, MO, USA) and Cayman Chemicals (Ann Arbor, MI, USA), respectively. After 48 h incubation, yeast cells were collected, washed twice in distilled water and kept at − 20 °C until further analysis.

### Fatty acid analysis of yeast

Total lipid was extracted from yeast and used to prepare fatty acid methyl esters (FAME) as described in detail previously [10]. FAME extraction, purification and analysis were performed as described by Li et al. [6]. Substrate fatty acid conversions from exogenously added PUFA substrates were calculated by the proportion of substrate fatty acid converted to a desaturated product as [product area/(product area + substrate area)] × 100 [21]. When appropriate, GC-MS was used to confirm the identity of the desaturation products [6].

## Results

### Gene orthologues of *fads1* and *fads2* emerged in the ancestor of vertebrates

To address the orthology of the identified Fads-like sequences an ML phylogenetic analysis was conducted with a total of 86 amino acid (aa) sequences, including species from cyclostomes (e.g. Arctic lamprey *L. camtschaticum*), tetrapods (e.g. amphibians, birds, mammals), Chondrichthyes (e.g. elephant shark *Callorhinchus milii*), ray-finned fishes (e.g. *P. senegalus*, *L. oculatus*, *A. japonica* and *P. buchholzi*), as well as Fads sequences from several protostomes (*Capitella teleta, Lingula anatina* and *Lottia gigantea*) and the invertebrate deuterostome *Saccoglossus kowalevskii*. The resulting phylogenetic tree displays two well-supported monophyletic clades, each containing vertebrate Fads1 and Fads2 sequences, respectively, with the invertebrate Fads appearing as an independent clade from both vertebrate Fads (Fig. 1). In the Fads1 group, in addition to the described gene orthologues from various lobe-finned fish (e.g. *Latimeria chalumnae*) and Chondrichthyes species (*C. milii* and *S. canicula*) [9], we find a putative Fads1 from the Arctic lamprey (*L. camtschaticum*), the Senegal bichir (*P. senegalus*), spotted gar (*L. oculatus*) and the teleost Japanese eel (*A. japonica*), but not in the Osteoglossomorpha or any other teleost examined (Fig. 1). A similar genetic distribution is observed in the Fads2 clade, with an orthologue of *fads2* found in the Arctic lamprey (Fig. 1). Moreover, Fads2 were also present in the pre-3R whole genome duplication (WGD) lineages such as Polypteriformes (*P. senegalus*) and Lepisosteiformes (*L. oculatus*) located at the ray-finned fish Fads2 clade. With the exception of *Arapaima gigas*, all Osteoglossomorpha species examined were found to possess two Fads2 (termed “a” and “b”), which are distributed among two well supported groups (0.9). Although, the branching tree pattern of the Osteoglossomorpha *fads2* gene duplicates could be indicative of an origin related with the teleost-specific 3R WGD, the absence of synteny precludes further analysis.

### Newly cloned *fads1* and *fads2* exhibit conserved Δ5 and Δ6 signature residues

**Fig. 2:**
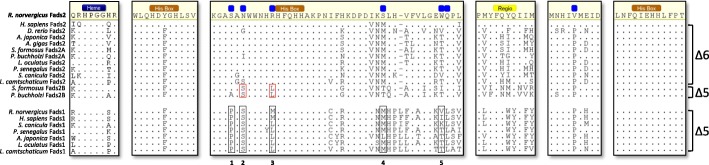
Sequence alignment of FADS1 and FADS2 amino acid sequences. Orange boxes correspond to the conserved histidine boxes, the yellow box indicates residues proposed to be involved in substrate specificity [27], and blue boxes indicate residues replaced in rat FADS2 Δ6 desaturase to obtain Δ5 activity [27]. The heme binding motif HPGG is also shown

To further characterise the newly identified Fads sequences, we cloned the full open reading frame (ORF) of each gene and performed Pfam searches of the deduced amino acid (aa) sequences. All the sequences presented the characteristic signature motifs of Fads, namely the heme binding motif (HPGG) and three histidine boxes HXXXH, HXXHH and QXXHH [22, 23] (Fig. 2, brown boxes). Next, we searched for critical aa residues that have been recently demonstrated to account for Δ5 (NP_445897.2) and Δ6 (NP_112634.1) desaturase activities in rat FADS enzymes [24]. Regarding Fads1, the analysed fish species preserved an overall Δ5 pattern with a conserved methionine (M) to leucine (L) substitution (Fig. 2, black box 3), possibly presenting no impact on the substrate specificity given the comparable biochemical properties of these residues. An additional valine (V) residue (Fig. 2, black box 5), also suggested to determine substrate selectivity in rat Fads1 [24], was not conserved in the analysed fish sequences, nor in the human sequence. Curiously, the *fads2b* sequences from the Osteoglossomorpha *P. buchholzi* and *S. formosus* presented key residues for Δ5 function, conserved with rat Fads1, serine (S) (Fig. 2, red box 2) and M/L (Fig. 2, red box 3). In contrast, the fads2a of *P. buchholzi* and *S. formosus* showed typical Δ6 residues (Fig. 2).

### Functional analysis indicates conserved and derived fads activities in various vertebrate species

Functional analysis of the newly isolated Fads was carried out by the heterologous expression of the ORF in the yeast *S. cerevisiae* grown in the presence of potential exogenously added polyunsaturated fatty acid (PUFA) substrates. Inactivity towards PUFA in control (untransformed) yeast was confirmed by fatty acid profiles characterised by major endogenous yeast fatty acids (16:0, 16:1n-7, 18:0 and 18:1n-9) and unmetabolised exogenously added PUFA (data not shown) [25]. Transgenic yeast expressing *fads1* from all species examined (*L. camtschaticum*, *P. senegalus*, *L. ocelatus* and *A. japonica*) showed Δ5 desaturase activity towards 20:4n-3 and 20:3n-6 since they were able to produce 20:5n-3 and 20:4n-6, respectively (Table 1 and Additional file 2). Moreover, yeast expressing *fads2* from *L. camtschaticum*, *P. senegalus*, *L. oculatus* and *P. buchholzi* (*Fads2a*) showed ∆6 activity, desaturating 18:3n-3 to 18:4n-3 and 18:2n-6 to 18:3n-6 (Table 1 and Additional file 2). In the vast majority of cases, Fads2 exhibited Δ8 desaturase capability as they were able to desaturate 20:2n-6 and 20:3n-3 to 20:3n-6 and 20:4n-3, respectively (Table 1 and Additional file 2), a feature previously confirmed in Fads2 of *A. japonica* [26]. Interestingly, transgenic yeast expressing *fads2b* of the African butterfly fish, *P. buchholzi*, showed neither Δ6 nor Δ8 desaturase activity but, as described above for Fads1 enzymes, a clear Δ5 desaturase profile, biosynthesising 20:4n-6 (ARA) and 20:5n-3 (EPA) from 20:3n-6 and 20:4n-3, respectively. No Δ4 desaturase activity was detected in any of the assayed Fads, which is in agreement with none of the sequences possessing the key aa residues responsible for Δ4 function (Fig. 2, yellow box) [27].

**Table 1:** Functional characterisation of isolated Fads enzymes

FA Substrate	FA Product	% Conversion										
		Lca Fads1	Lca Fads2	Loc Fads1	Loc Fads2	Pse Fads1	Pse Fads2	Aja Fads1	Aja^a^ Fads2	Pbu Fads2A	Pbu Fads2B	Activity
18:3n-3	18:4n-3	n.d.	6.6	n.d.	32.4	n.d.	37.1	n.d.	64.3	77.4	n.d	Δ6
18:2n-6	18:3n-6	n.d.	2.0	n.d.	15.6	n.d.	20.6	n.d.	20.7	42.7	n.d	Δ6
20:3n-3	20:4n-3	n.d.	0.7	n.d.	4.1	n.d.	11.0	n.d.	6.0	18.4	n.d	Δ8
20:2n-6	20:3n-6	n.d.	n.d.	n.d.	1.5	n.d.	3.6	n.d.	5.4	7.0	n.d.	Δ8
20:4n-3	20:5n-3	6.0	n.d.	3.0	n.d.	56.1	n.d.	58.1	n.d.	n.d.	14.4	Δ5
20:3n-6	20:4n-6	5.5	n.d.	2.9	n.d.	48.3	n.d.	33.2	n.d.	n.d.	11.7	Δ5
22:5n-3	22:6n-3	n.d.	n.d.	n.d.	n.d.	n.d.	n.d.	n.d.	n.d.	n.d.	n.d.	Δ4
22:4n-6	22:5n-6	n.d.	n.d.	n.d.	n.d.	n.d.	n.d.	n.d.	n.d.	n.d.	n.d.	Δ4
Overall activity		Δ5	Δ6/Δ8	Δ5	Δ6/Δ8	Δ5	Δ6/Δ8	Δ5	Δ6/Δ8	Δ6/Δ8	Δ5	

The conversions were calculated according to the formula [product area/(product area + substrate area) × 100]

*Lca Lethenteron camtschaticum*, *Loc Lepisosteus oculatus*, *Pse Polypterus senegalus*, *Aja Anguilla japonica*, *Pbu Pantodon buchholzi*, *n.d.* indicates not detected

^a^Data collected from [6]

## Discussion

Gene duplication has long been recognised as a decisive contributor in the shaping vertebrate genomes, providing spare genetic material for adaptive evolution, mutation, and genetic drift [28–31]. Yet, degeneration and loss are the most common fates encountered with duplicate genes [3], with significant gene loss occurring shortly after WGD episodes [28, 29, 32–36]. To evaluate the impact of gene duplication and loss on LC-PUFA biosynthesis in vertebrates, we investigated the genetic repertoire and function of *fads* genes in species placed at key phylogenetic points, namely the transition from jawless to jawed vertebrates, and the pre/post 3R period in the evolution of the ray-finned fish.

**Fig. 3:**
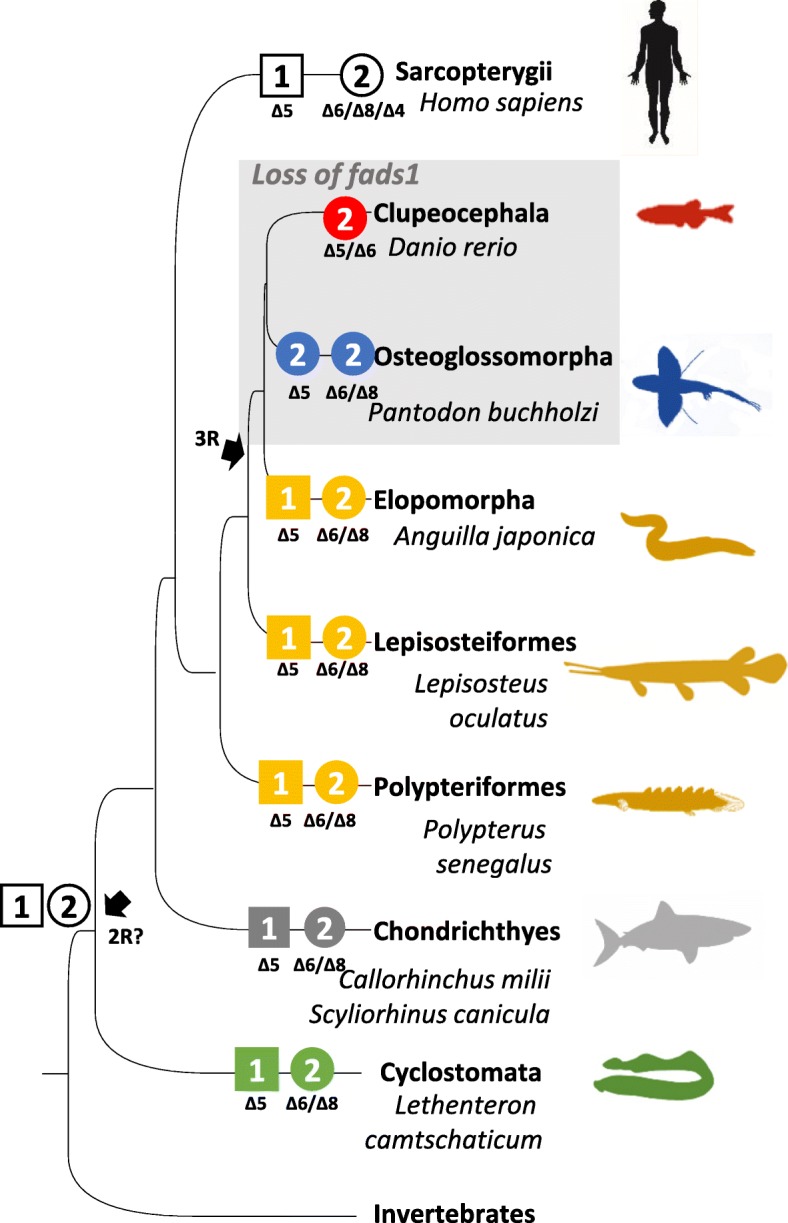
Evolutionary history and distribution of Fads1 and Fads2 in vertebrates, combined with the corresponding desaturase activities. Squares correspond to *fads1* circle to *fads2*, black arrows indicate the approximate timing of whole genome duplications in the ancestral vertebrate (2R) and the teleost specific duplication (3R), grey square highlights lineages lacking *fads1*

Sequence and phylogenetic data revealed that *fads1* and *fads2* orthologues are present in the genome of the Arctic lamprey (*L. camtschaticum*), supporting the hypothesis that *fads1* and *fads2* originated most likely thorough a tandem gene duplication in the vertebrate ancestor (Fig. 3) [9]. Additionally, we found that *fads1* and *fads2* are also retained in ray-finned fish such as the Senegal bichir (*P. senegalus*) and the spotted gar (*L. oculatus*), which diverged prior to the Teleostei specific 3R WGD (Fig. 3). Unexpectedly, a *fads1* orthologue was also identified in species such as the Japanese eel (*A. japonica*) and the Indo-Pacific tarpon (*Megalops cyprinoides*) belonging to Elopomorpha, a group that diverged after the 3R WGD [15, 16]. These results indicate that *fads1,* previously hypothesised to be lost in Teleostei [3, 9, 11], is actually retained in some Teleostei such as the Elopomorpha (Fig. 3). We were unable to recover a *fads1* orthologue in *P. buchholzi* nor in any of the other Osteoglossomorpha species analysed, indicating that this gene is likely lost in this lineage. Further, in silico search for *fads1* in relevant genome databases of representatives of Euteleosteomorpha or Otomorpha lineages suggest an absence of *fads1* in Clupeocephala, which further supports Elomorpha as the only Teleostei retaining a functional *fads1*. Yet, all Fads1 desaturases characterised in the present work (*L. camtschaticum*, *P. senegalus*, *L. ocelatus* and *A. japonica*) exhibit ∆5 desaturase activity in agreement with the previous functional assessments in Sarcopterygii such as *H. sapiens* FADS1 [37] and chondrichthyan Fads1 [9].

In contrast to the lack of *fads1* within most Teleostei, phylogenetic analysis confirmed that *fads2* is ubiquitously present across the entire vertebrate clade, with exceptions represented by teleosts lacking *fads*-like orthologues in their genomes, namely *Takifugu rubripes* and *T. nigroviridis* [3, 38]. Thus, vertebrate Fads2 includes not only those previously reported from numerous species within Teleostei, Sarcopterygii and Chondrichthyes [3], but also the presently functionally characterised Fads2 from Cyclostomata (Arctic lamprey), Polypteriformes (Senegal bichir), Lepisosteiformes (spotted gar), Elopomorpha (Japanese eel) and Osteoglossomorpha (African butterfly fish). Interestingly, we found that Osteoglossomorpha species presented two copies of *fads2* that clustered into two separate groups in the phylogenetic analysis and therefore suggesting a potential origin dating to the 3R WGD. With regards to their functional characterisation, the African butterfly fish *fads2b* demonstrated that this gene did not encode a ∆6 desaturase, but rather a desaturase that showed ∆5 activity (Table 1). A similar functionalisation scenario among teleost Fads2 was also suggested in some salmonids such as Atlantic salmon (*Salmo salar*) and rainbow trout (*Oncorhynchus mykiss*), with the acquisition of ∆5 activity occurring in one of the several *fads2* copies that arose from tandem gene duplication [3, 39, 40]. In fact, *fads* gene duplication appears to be a frequent event in vertebrate evolution, with most mammals possessing an additional gene, *Fads3* [41–43], and the well-established *fads2* duplication occurred in Teleostei [6, 10, 11, 39, 44]. Gene duplication is often followed by low purifying selection; thus, the presence of Δ5 functionalised Fads2b in Teleostei, such as osteoglossomorpha, is consistent with subfunctionalisation and/or neofunctionalisation processes occurring rapidly after gene duplication [35, 45].

The conversion of a *fads2*Δ6 ancestor, as deduced from the data from the Senegal bichir and the spotted gar, into a *fads2*Δ5 in African butterflyfish (*fads2b*) may be viewed as a mechanism to overcome the bottleneck generated by the loss of *fads1*Δ5 in most Teleostei that would otherwise be unable to biosynthesise essential fatty acids such as EPA and ARA. Yet, such a constraint triggered distinct evolutionary routes. In addition to the abovementioned case in salmonids, whereby acquisition of Δ5 desaturase occurred in one of the several Fads2 copies, in other species such as *D. rerio* [10] with one *fads2*, the acquisition of Δ5 desaturase activity has been accompanied by retention of Δ6 activity, thus resulting in desaturases with dual (or bifunctional) Δ6Δ5 activities (Fig. 3), which have also been found in other Teleostei [11, 13, 46, 47]. Further functionalisation cases amongst teleost Fads2 include the presence of Δ4 desaturases, often co-existing with Δ5 desaturase activity within the same enzyme [6, 11, 12]. Still, the majority of Teleostei Fads2 are primarily Δ6 desaturases without Δ5 or Δ4 desaturase activities [3]. Interestingly, the functional characterisation data confirm that, with the exception of the African butterfly fish Fads2b (Δ5 desaturase), all the presently analysed Fads2 showed capability for Δ8 desaturation, an intrinsic enzymatic ability within vertebrate Fads2 and hence not regarded as a functionalisation case [42, 44].

The LC-PUFA biosynthetic pathway is punctuated by alternate steps of fatty acid elongation and desaturation therefore, a comprehensive interpretation of the evolution of this key metabolic pathway in vertebrates can be only fully attained if the repertoire and function of Elovl enzymes is also considered. Similarly to Fads1 and Fads2, Elovl2 and Elovl5, major elongation enzymes involved in LC-PUFA biosynthesis [48], emerged in vertebrate ancestry possibly as a consequence of genome duplications, and as demonstrated by the existence of both orthologues in Arctic lamprey and elephant shark [49]. Therefore, the ability to biosynthesise EPA and ARA was present in vertebrates such as lamprey. For DHA biosynthesis, two consecutive elongation reactions from EPA are required to produce 24:5n-3, which is then Δ6 desaturated to 24:6n-3 before it is partly β-oxidised to DHA (22:6n-3) [4]. While the Δ6 desaturation activity towards 24:5n-3 has been shown to be an intrinsic characteristic of non-Δ4 Fads2 desaturases and it is widely distributed from basal gnathostomes to recently emerged Teleostei [7], the elongation capacity towards C_22_ substrates such as 22:5n-3 hinders the “*Sprecher pathway*” in cyclostomes since the Arctic lamprey Elovl2 could not elongate 22:5n-3 [49]. In pre 3R WGD Teleostei, along with Fads1Δ5 and Fads2Δ6 reported here, it is likely that Elovl2 and Elovl5 (present in at least spotted gar, Elovl2- XP_015210453.1 and Elovl5- XP_006638754.1) constitute a complete LC-PUFA biosynthesis cascade, as demonstrated in chondrichthyans [49], a scenario that can be also postulated for post 3R WGD Teleostei such as Elopomorpha. Yet, other 3R WGD lineages including cyprinids, siluriformes and salmonids have Elovl2 and Elovl5 [13, 25, 50–52] and, while lacking Fads1 gene orthologues, complete the LC-PUFA biosynthetic cascade with functionalised Fads2Δ5 desaturase (e.g. *D. rerio*). Consequently, a key event in Teleostei evolution included also that of Elovl2, crucial for elongation reactions involved in DHA biosynthesis, although Elovl4 has been seen to partly compensate such gene loss in Teleostei [53]. The parallel, and contingent, evolution of two separate gene families, Fads and Elovl, in vertebrate ancestry, allowed for the combination of pre-existing metabolic islands into an integrated and functional enzymatic cascade, which was indispensable for adaptive paths with respect to LC-PUFA habitat availability.

## Conclusion

In the present study, we established that the fatty acid desaturation dependent of *fads1Δ5* and *fads2Δ6* orthologues arose before vertebrate radiation, as deduced from the Arctic lamprey data. Additionally, *fads1* was retained in pre 3R WGD lineages such as Polypteriformes (Senegal bichir) and Lepisosteiformes (spotted gar) and post 3R WGD Elopomorpha (Japanese eel) but was probably lost in the Osteoglossomorpha and Clupeocephala. Moreover, in Osteoglossomorpha the two reported *fads2* genes exhibited the expected Δ6 desaturase activity, while the second *fads2* paralogue (*fads2b*) had Δ5 activity. This observation supports the existence of alternative evolutionary routes that mitigate the loss of the canonical *fads1Δ5* in Teleostei and puts forward Osteoglossomorpha Fads2 as, possibly, the most ancient representative of Fads2 plastic functionalisation.

## Additional files

Additional file 1: **Table S1.** Primer sets, corresponding PCR conditions. (DOCX 23 kb)

Additional file 2: **Figure S1.** GC-MS Chromatograms of Fads functional characterisation. (PDF 1338 kb)

## Abbreviations

2R WGD: Vertebrate whole genome duplication, or two rounds of whole genome duplication
3R WGD: Teleost specific whole genome duplication
ALA: α-linolenic acid
ARA: Arachidonic acid
DHA: Docosahexaenoic acid
EFA: Essential fatty acid
Elovl: Fatty acid elongase
EPA: Eicosapentaenoic acid
Fads: Fatty acid desaturase
FAME: Fatty acid methyl esters
GC-MS: Gas chromatography–mass spectrometry
LA: Linoleic acid
LC-PUFA: Long-chain polyunsaturated fatty acids
ORF: Open reading frame
PCR: Polymerase chain reaction
PUFA: Polyunsaturated fatty acids
SRA: Sequence read archive
WGD: Whole genome duplication
