# Supplementary material for: Retention of fatty acyl desaturase 1 (fads1) in Elopomorpha and Cyclostomata provides novel insights into the evolution of long-chain polyunsaturated fatty acid biosynthesis in vertebrates
Source: BMC Evol Biol. 2018 Oct 19;18:157. doi: 10.1186/s12862-018-1271-5 (PMC6194568; doi:10.1186/s12862-018-1271-5)
Supplement: Supplementary file 1 — Table S1. Primer sets, corresponding PCR conditions. (DOCX 23 kb) [file 12862_2018_1271_MOESM1_ESM.docx]

**Additional file 1**

**Table S1.** Primer sets, corresponding PCR conditions.

|  | PCR details |  | Primer sequence | Initial denaturation | Cycles | Denaturation | TM | Extension | Final extension |
| --- | --- | --- | --- | --- | --- | --- | --- | --- | --- |
| *Polypterus senegalus* | Phusion Flash High-Fidelity PCR Master Mix | ***Fads1*** | **Fw:** ccc**GGTACC**ATGGAGGATGAAACAAAAGATAAAA | 98ºC /10s | 45 | 98ºC /1s | 61ºC/5s | 72ºC/21s | 72ºC/1min |
|  |  |  | **Rv:** CCC**TCTAGA**TCACTTATGCAGGTAGGCGTC |  |  |  |  |  |  |
|  |  | ***Fads2*** | **Fw:** ccc**GGTACC**CCTAAAATGGGGAAAGGTGG | 98ºC /10s | 35 | 98ºC /1s | 61ºC/5s | 72ºC/21s | 72ºC/1min |
|  |  |  | **Rv:** ccc**tctaga**GTTTCTCTCTTTCTTACTTGTTAAG |  |  |  |  |  |  |
| *Lepisosteus oculatus* | Phusion Flash High-Fidelity PCR Master Mix | ***Fads1***  ***Long*** | **Fw**: CCC**GGATCC**AGGATGGGCGCAGGCGCAGA | 98ºC /10s | 40 | 98ºC /1s | 69ºc/5s | 72ºC/20s | 72ºC/1min |
|  |  |  | **Rv**: CCG**TCTAGA**TCACCTGTGCAGGTAGGCATCAAGC |  |  |  |  |  |  |
|  | Phusion Flash High-Fidelity PCR Master Mix+ 3%DMSO | ***Fads2*** | **FW:** CCC**GGTACC**ACAATGGGTGGGGGGGGCCAGC | 98ºC /30s | 40 | 98ºC /1s | 68ºc/5s | 72ºC/20s | 72ºC/30s |
|  |  |  | **RV:** CCC**TCTAGA**CCTATTTGTGGAGGTAGGCATCCA |  |  |  |  |  |  |
| *Pantodon buchholzi* | Phusion Flash High-Fidelity PCR Master Mix | ***Fads2a*** | **FW:** CCC**GGTACC**ATGGGAGGCGGTGGGCAGC | 98ºC /10s | 35 | 98ºC /1s | 65ºC/5s | 72ºC/20s | 72ºC/1min |
|  |  |  | **RV:** CCC**TCTAGA**TCACTTATGCAGGTAGGCATCCAG |  |  |  |  |  |  |
|  |  | ***Fads2b*** | **FW:** CCC**GGATCC**AATATGGGTGGTGGAGGACAGC | 98ºC /10s | 35 | 98ºC /1s | 65ºC/5s | 72ºC/20s | 72ºC/1min |
|  |  |  | **RV**: CCC**CTCGAG**TTACTTGTGAAGGTACGCATCCAG |  |  |  |  |  |  |
| *Anguilla japonica* | *Pfu* DNA polymerase | ***Fads1*** | **FW:**CCC**AAGCTT**AACATGAGCGCAGCAGAGAAG | 95ºC/2min | 35 | 95ºC /30s | 60ºC/30s | 72ºC/3min | 72ºC/5min |
|  |  |  | **RV:**CCG**TCTAGA**TCATTTGTGCAAGTAAGCATCCATC |  |  |  |  |  |  |
