# Supplementary material for: Retention of fatty acyl desaturase 1 (fads1) in Elopomorpha and Cyclostomata provides novel insights into the evolution of long-chain polyunsaturated fatty acid biosynthesis in vertebrates
Source: BMC Evol Biol. 2018 Oct 19;18:157. doi: 10.1186/s12862-018-1271-5 (PMC6194568; doi:10.1186/s12862-018-1271-5)
Supplement: Supplementary file 2 — Figure S1. GC-MS Chromatograms of Fads functional characterisation. (PDF 1338 kb) [file 12862_2018_1271_MOESM2_ESM.pdf]

Additional file 2

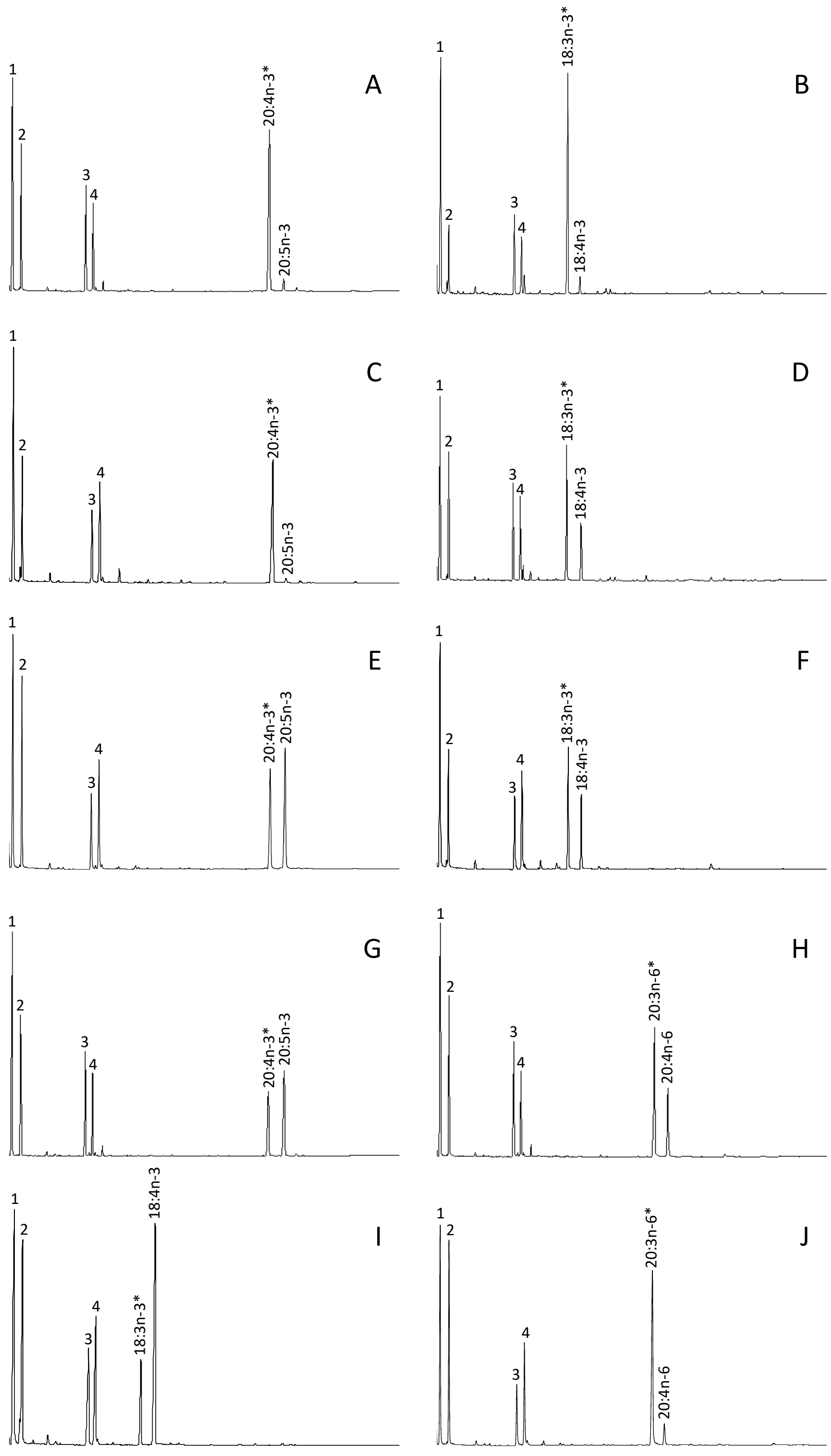

Figure S1. Chromatograms of fatty acid (FA) methyl esters from the transgenic yeast expressing *L. camtschaticum* Fads1 (A) and Fads2 (B), *L. oculatus* Fads1(C) and Fads2 (D), *P. senegalus* Fads1 (E) and Fads2 (F), *A. japonica* Fads1 (G, H), and *P. buchholzi* Fads2A (I) and Fads2B (J). All transgenic yeast were grown in the presence of an exogenously added FA substrates (indicated as "*" in all panels). Peaks 1–4 represent endogenous FA of the yeast, namely 16:0 (1), 16:1 isomers (2), 18:0 (3) and 18:1n-9 (4).
